# Qufeng Xuanbi Formula Ameliorates Airway Remodeling in Asthmatic Mice by Suppressing Airway Smooth Muscle Cell Proliferation through MEK/ERK Signaling Pathway

**DOI:** 10.1155/2022/1525110

**Published:** 2022-02-09

**Authors:** Bohan Wang, Lingling Tang, Suofang Shi, Ying Yang, Xianhong Sun, Xiaona Zhang, Chunyang Liu, Li Liu

**Affiliations:** Affiliated Hospital of Nanjing University of Chinese Medicine, Nanjing, Jiangsu 210029, China

## Abstract

Asthma is a common chronic respiratory disease. The Qufeng Xuanbi formula (QFXBF), a Chinese herbal decoction, has shown efficacy in the management of asthma. The purpose of this study was to investigate the potential therapeutic effects of QFXBF in the treatment of asthma both in vitro and in vivo. Platelet-derived growth factor (PDGF)-induced airway smooth muscle cell (ASMC) proliferation and MTT assays were used to explore the effects of QFXBF on the proliferation of ASMCs. Moreover, 40 female BALB/c mice were randomly divided into five groups: control group, ovalbumin (OVA) group, high QFXBF group, low QFXBF group, and dexamethasone (DEX) group (*n* = 8 per group). A mouse allergic asthma model was established using the intranasally administered OVA sensitization method. Morphological changes in the lung tissue were examined by hematoxylin and eosin (H&E) staining and Masson's trichrome staining. Finally, the protein expression of alpha-smooth muscle actin (*α*-SMA), proliferating cell nuclear antigen (PCNA), phospho-mitogen-activated protein kinase (p-MEK1/2), mitogen-activated protein kinase (MEK1/2), phospho-extracellular signal-regulated kinases (p-ERK1/2), and extracellular signal-regulated kinases (ERK1/2) in ASMCs and lung tissue were determined by western blotting and immunofluorescent staining assays. PDGF significantly increased the viability of ASMCs. Compared with mice in the control group, the airway walls and airway smooth muscle of mice in the OVA group were thickened, and the number of inflammatory cells around the bronchus significantly increased. Moreover, the administration of QFXBF markedly inhibited the proliferation of ASMCs and alleviated the pathological changes induced by OVA. Furthermore, the protein expressions of p-ERK1/2, p-MEK1/2, PCNA, and *α*-SMA were significantly increased in OVA-treated mice and PDGF-treated ASMCs. Finally, treatment with QFXBF also significantly decreased the protein expression of p-ERK1/2, p-MEK1/2, *α*-SMA, and PCNA. QFXBF inhibited the proliferation of ASMCs by suppressing MEK/ERK signaling in PDGF-induced ASMCs and OVA-induced mice.

## 1. Introduction

Asthma is a chronic respiratory disease that affects nearly 400 million individuals worldwide. Increasing evidence suggests that asthma has become a global healthcare issue that significantly affects the quality of life of patients while also inducing a massive increase in healthcare burden [1]. Chronic airway inflammation and airway remodeling are key pathological changes in the pathogenesis of asthma [2]. Airway remodeling is the main factor regulating the progression of asthma, which leads to an incomplete reversible obstruction of airflow [3]. At present, glucocorticoid drugs are still the first-line medication for the management of asthma; however, the safety issue remains to be solved, and whether glucocorticoid medications could fundamentally reduce the process of airway remodeling remains further investigation [4, 5]. Therefore, it is vital to explore the pathogenesis of airway remodeling in asthma and develop potential antiasthma therapies with improved therapeutic efficacy.

Airway remodeling caused by abnormal proliferation of airway smooth muscle cells (ASMCs) is a typical pathological feature of asthma [6, 7]. Previous studies have suggested that multiple factors can regulate the proliferation of AMSCs; for example, it has been reported that the mitogen-activated protein kinase (MEK)/extracellular signal-regulated kinase (ERK) signaling pathway plays a key role in the development of asthma [8]. ERK is widely distributed in differentiated cells [9], and ERK is known to participate in multiple cellular activities, including the differentiation, secretion, and proliferation of smooth muscle cells [10] and tumor cells [11]. Studies have shown that phosphorylated ERK can regulate the expression of various growth factors and inflammatory mediators, which further inhibit the overproliferation of ASMCs and consequently lead to airway remodeling in asthma [12]. On the other hand, inhibition of the MEK/ERK signaling pathway can suppress the overproliferation and migration of endothelial cells in vitro [13, 14]. Therefore, exploring the potential roles of the MEK/ERK signaling pathway in the pathogenesis of asthma may provide a new method for the treatment of asthma.

The Qufeng Xuanbi formula (QFXBF) has been approved for hospital prescription in the Affiliated Hospital of Nanjing University of Chinese Medicine (Nanjing, Jiangsu, China) and has been used for the treatment of asthma during the past 30 years due to its high efficiency in the management of refractory asthma. Previous studies have shown that QFXBF can reduce eosinophil (EOS) infiltration, decrease airway inflammation, and consequently inhibit the occurrence and development of asthma by regulating the imbalance of Th1/Th2 [15]. However, the pharmacological effects of QFXBF on the MEK/ERK signaling pathway during airway remodeling require further investigation. Therefore, we treated ASMCs with PDGF to create a proliferation cell model in vitro and established an asthmatic mouse model by OVA stimulation. The objective of this study was to investigate the effects of QFXBF on airway remodeling in asthma by modulating the MEK/ERK signaling pathway.

## 2. Material and Methods

### 2.1. Chemicals and Reagents

Platelet-derived growth factor (PDGF) was purchased from Proteintech (Wuhan, China); U0126 was provided by Selleck Chemicals (Shanghai, China); fetal bovine serum (FBS), RPMI-1640, and penicillin-streptomycin solution were purchased from Gibco (Thermo Fisher Scientific, Waltham, MA); ovalbumin (OVA) was obtained from Sigma-Aldrich (Saint Louis, MO, United States); and dexamethasone (DEX) was purchased from Xianju Pharmaceuticals (Zhejiang, China). p-ERK1/2, ERK1/2, and HRP-conjugated secondary antibodies were purchased from Cell Signaling Technology (Beverly, MA, USA); p-MEK1/2, MEK1/2, and *β*-actin antibodies were obtained from Affinity (Changzhou, China); PCNA, *α*-SMA, and GAPDH antibodies were purchased from Proteintech (Wuhan, China); and RIPA lysis buffer and BCA protein assay kit were obtained from Beyotime (Shanghai, China).

### 2.2. Preparation of QFXBF

QFXBF is composed of Belamcandae rhizoma 10 g, Ephedrae herba 5 g, Semen Armeniacae Amarum 10 g, Glycyrrhizae radix et Rhizoma 5 g, Pheretima 10 g, Trichosanthis Fructus 10 g, Allium macrostemon Bunge 10 g, Bombyx batryticatus 10 g, Kadsura Pepper Stem 15 g, and Cnidii Fructus 15 g. All herbs were obtained from the Affiliated Hospital of Nanjing University of Chinese Medicine (Nanjing, Jiangsu, China). The QFXBF decoction was prepared according to a conventional method. All of the herbs were impregnated in 1,000 ml water for 1 h, boiled for 45 minutes, and then the solution was collected; then, 500 ml of water was added to the residual liquid and boiled for further 45 min; the two extracts were then filtered, concentrated to 1 g/ml, and stored at 4°C. Chromatographic analysis of the QFXBF sample showed that the active ingredients in this prescription included tectorigenin, glycyrrhizic acid, amygdalin, and osthole [15].

### 2.3. Cell Culture and Treatment

Mouse ASMCs were purchased from the Chinese Academy of Sciences (Shanghai, China). ASMCs were cultured in RPMI-1640 medium supplemented with 10% FBS and penicillin-streptomycin and cultured in an incubator at 37°C with 5% CO2. ASMCs were seeded at 2×105/well in a 6-well plate. After the cells had adhered, 10 ng/ml PDGF was added to the cell culture medium. Treatment with QFXBF (2 mg/ml, 4 mg/ml, and 8 mg/ml) or U0126 (10 *μ*mol/L) begins after the induction of PDGF and lasted for an additional 48 h before harvesting.

### 2.4. Cell Proliferation Assay

Cell proliferation was determined using the MTT cell viability assay kit (Beyotime Biotechnology, Shanghai, China). ASMCs were seeded onto 96-well plates at 8,000 cells/well, and after treatment, 15 *μ*L MTT (5 mg/mL) was added to each well and incubated for 4 h. The supernatant was then removed, and 150 *μ*L dimethyl sulfoxide (DMSO) was added to each well. Cell viability was determined by measuring the absorbance of each well at 490 nm using an ELX800 automatic microplate reader (BioTek, USA).

### 2.5. Animals and Treatment

The animal protocol was approved by the Animal Care and Use Committee of the Affiliated Hospital of Nanjing University of Chinese Medicine (2021DW-07-02, Nanjing, China). Forty female BALB/c mice (6–8 weeks old, weighing 18–22 g) were purchased from Shanghai SLAC Laboratory Animal Company (Shanghai, China) and housed in the Animal Laboratory of the Affiliated Hospital of Nanjing University of Chinese Medicine (Nanjing, China). After one week of adaptive feeding, the mice were randomly divided into five groups: control, OVA, high QFXBF, low QFXBF, and DEX groups. On days 0 and 14, mice in the OVA, high QFXBF, low QFXBF, and DEX groups were sensitized via intraperitoneal injection of 100 µg of OVA combined with aluminum hydroxide. On days 14, 25, 26, and 27, every mouse in the aforementioned groups was intranasally administered with 50 µL of OVA. Normal saline was used in the control group following the same procedure. Between days 14 and 27, mice in the high QFXBF group (25 g/kg) and low QFXBF group were intragastrically administered QFXBF (12.5 g/kg) once/day, while mice in the DEX group were intragastrically administered with 2 mg/kg DEX once/day, and mice in the saline and OVA groups received normal saline (0.5 mL). On day 28, the mice were sacrificed, and the pathophysiological and immunological features of asthma were determined.

### 2.6. Histopathology

Lung tissues were fixed with 10% formalin liquid, and the fixed sections were embedded in paraffin, sectioned, and stained with H&E and Masson. Tissue sections were visualized using light microscopy at a magnification of x 400. The bronchial basement membrane perimeter (Pbm), total bronchial area (WAt1), bronchial lumen area (WAt2), area of the inner trachea at the outer edge of smooth muscle (WAm1), and endotracheal area at the inner edge of smooth muscle (WAm2) were measured using Image-Pro Plus software. WAt1-WAt2 and WAm1-WAm2 are used to denote bronchial wall area (WAt) and bronchial smooth muscle area (WAm), respectively, and normalized to Pbm. The bronchial wall thickness (WAt/Pbm) and bronchial smooth muscle thickness (WAm/Pbm) were calculated for indicating the degree of airway remodeling as previously described [16, 17].

### 2.7. Western Blot Assay

Total proteins were extracted from lung tissues and ASMCs, and protein concentrations were quantified using the bicinchoninic acid (BCA) protein assay kit. Equal amounts of protein samples were separated by 10% SDS-PAGE (80 V, 30 min and then 120 V, 60 min), then transferred onto a polyvinylidene fluoride (PVDF) membrane (250 mA, 90 min), and then blocked with 5% bovine serum albumin (BSA) for 60 min at room temperature. The membranes were then incubated with primary antibodies (anti-PCNA (1 : 2,000 dilution), anti-*α*-SMA (1 : 1,000 dilution), anti-p-MEK1/2 (1 : 1,000 dilution), anti-MEK1/2 (1 : 500 dilution), anti-p-ERK1/2 (1 : 1,000 dilution), and anti-ERK1/2 (1 : 1,000 dilution)) overnight at 4°C. On day 2, the membranes were incubated with fluorescence-conjugated secondary antibodies (1 : 3,000 dilution). ECL luminescent liquid was used for visualization. The results were analyzed and quantified using Image Lab software (Bio-Rad, USA).

### 2.8. Immunofluorescence Assay

After fixing in immunol staining fix solution, the cells were permeabilized with 0.3% Triton X-100 in PBS 3 times (5 min each) and blocked with 5% BSA for 30 min at room temperature. The cells were then incubated with primary antibodies (anti-PCNA (1 : 50 dilution) and anti-*α*-SMA (1 : 100 dilution)) overnight at 4°C. On day 2, cells were washed with PBS 3 times, then incubated with the secondary antibodies (1 : 100 dilution) for 60 min at room temperature, washed with PBS for 3 min, and then stained with 4′, 6-diamidino-2-phenylindole (DAPI). Finally, the cells with immunofluorescence staining were visualized using a fluorescence microscope (NIKON, Japan).

### 2.9. Statistical Analysis

Data are expressed as the mean ± standard deviation. Statistical analyses were performed using SPSS 23.0. Differences among multiple groups were analyzed by one-way ANOVA, and statistical significance was set at *p* < 0.05.

## 3. Results

### 3.1. QFXBF Inhibited the Hyperproliferation of ASMCs Induced by PDGF

**Figure 1:**
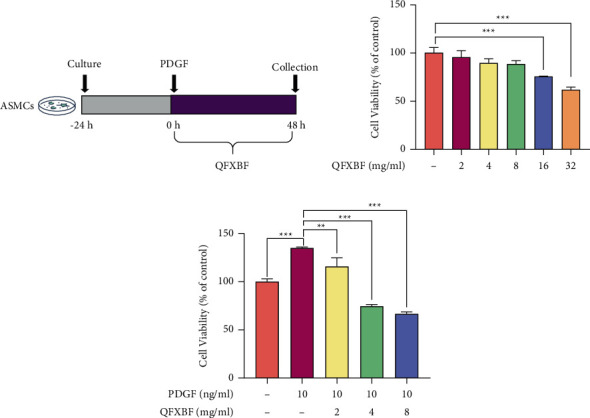
QFXBF inhibited the hyperproliferation of ASMCs induced by PDGF. (a) The protocol of current work. (b) MTT assay has been applied to determine the cytotoxic effect of QFXBF on the viability of ASMCs. (c) Cell viability was detected by MTT assay. All data were presented as means ± SD.  ^*∗*^*p* < 0.05,  ^*∗*^ ^*∗*^*p* < 0.01, and  ^*∗*^ ^*∗*^ ^*∗*^*p* < 0.001.

To verify the inhibitory effects of QFXBF on the hyperproliferation of ASMCs, PDGF was used to stimulate ASMCs in order to build an overproliferation cell model (Figure 1(a)). The cytotoxic effects of QFXBF on ASMCs were examined by the MTT assay. ASMCs were treated with QFXBF at different concentrations (2–32 mg/mL) for 48 h, and the maximum nontoxic concentration of QFXBF was 8 mg/ml (Figure 1(b)). Moreover, as shown in Figure 1(c), compared with the control group, treatment of PDGF significantly promoted the proliferation of ASMCs, while the administration of QFXBF markedly reduced the proliferation of ASMCs in a dose-dependent manner compared with the PDGF group.

### 3.2. QFXBF Inhibited the Expressions of *α*-SMA and PCNA in ASMCs

**Figure 2:**
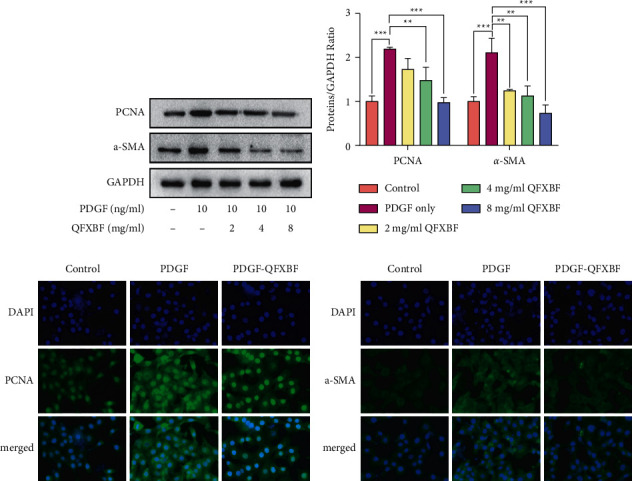
QFXBF inhibited the expression of *α*-SMA and PCNA in ASMCs. (a) Expressions of PCNA and *α*-SMA protein in ASMCs via western blot. (b) Quantification of WB results in (a). (c) Expression of PCNA (c) and *α*-SMA (d) in ASMCs via immunofluorescence staining. All data are presented as means ± SD.  ^*∗*^*p* < 0.05,  ^*∗*^ ^*∗*^*p* < 0.01, and  ^*∗*^ ^*∗*^ ^*∗*^*p* < 0.001.

Overexpression of PCNA and *α*-SMA is a significant hallmark of ASMC proliferation; therefore, the protein expression of PCNA and *α*-SMA was detected by western blot assay. As shown in Figures 2(a) and 2(b), PDGF induced significant upregulation of PCNA and *α*-SMA in ASMCs compared with the control group, while administration of QFXBF reduced the expression of PCNA and *α*-SMA. In addition, immunofluorescence staining was performed to examine the expression of *α*-SMA and PCNA (Figures 2(c)–2(d)), and the results were similar to those of the western blot.

### 3.3. PDGF-Induced Hyperproliferation of ASMCs via Regulating MEK/ERK Signaling Pathway

**Figure 3:**
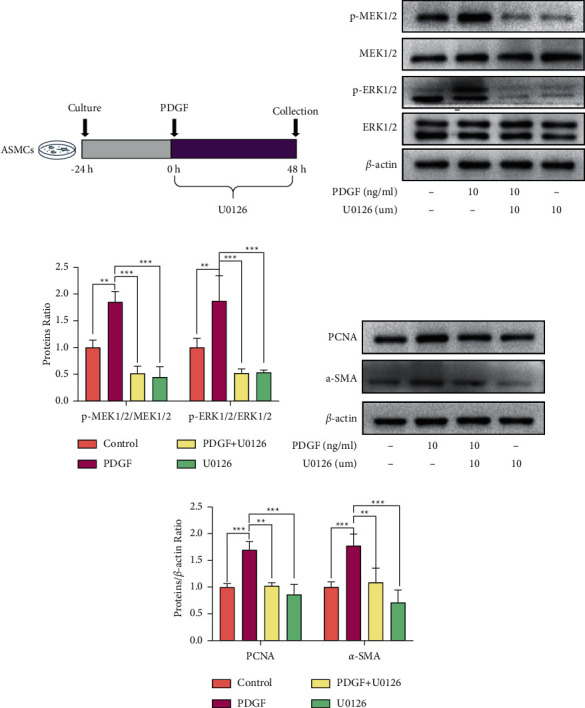
PDGF-induced hyperproliferation of ASMCs through regulating the MEK/ERK signaling pathway. (a) Experimental protocol of current work. (b) Protein expressions of p-MEK1/2, MEK1/2, p-ERK1/2, and ERK1/2 in ASMCs after PDGF or U0126 administration via western blot methods. (c) Quantification of results in (b). (d) Protein expressions of PCNA and *α*-SMA in ASMCs after PDGF or U0126 administration by western blot methods. (e) Quantification of results in (d). All data are presented as means ± SD.  ^*∗*^*p* < 0.05,  ^*∗*^ ^*∗*^*p* < 0.01, and  ^*∗*^ ^*∗*^ ^*∗*^*p* < 0.001.

The experimental protocol is shown in Figure 3(a). As shown in Figures 3(b)–3(c), the expressions of p-MEK1/2 and p-ERK1/2 were significantly increased in the PDGF-induced hyperproliferation model compared to that in the control; on the other hand, U0126, an inhibitor of the MEK/ERK signaling pathway, markedly reduced the phosphorylation of MEK and ERK induced by PDGF and also inhibited the proliferation of ASMCs by inhibiting the expression of *α*-SMA and PCNA (Figures 3(d)–3(e)).

### 3.4. QFXBF Inhibits PDGF-Induced Hyperproliferation of ASMCs via Regulating MEK/ERK Signaling Pathway

**Figure 4:**
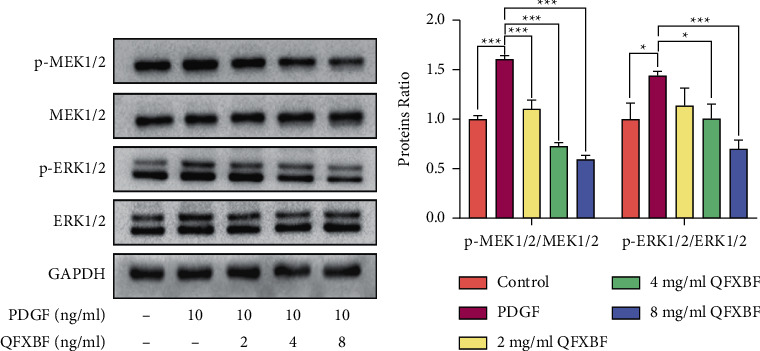
QFXBF inhibits PDGF-induced hyperproliferation of ASMCs via regulating the MEK/ERK signaling pathway. (a) Expressions of p-MEK1/2, MEK1/2, p-ERK1/2, and ERK1/2 in ASMCs via western blot method. (b) Quantification of results in (a). All data are presented as means ± SD.  ^*∗*^*p* < 0.05,  ^*∗*^ ^*∗*^*p* < 0.01, and  ^*∗*^ ^*∗*^ ^*∗*^*p* < 0.001.

Furthermore, we explored the effects of QFXBF on the activation of MEK/ERK signaling in PDGF-induced hyperproliferation of ASMCs by western blotting. As shown in Figures 4(a) and 4(b), the expressions of p-MEK1/2 and p-ERK1/2 in ASMCs were significantly upregulated in the PDGF-induced hyperproliferation model compared to the control group. In contrast, QFXBF treatment significantly reduced the phosphorylation of p-MEK1/2 and p-ERK1/2 in a dose-dependent manner.

### 3.5. QFXBF Alleviated the Inflammation and Airway Remodeling in OVA-Induced Asthmatic Mice Models

**Figure 5:**
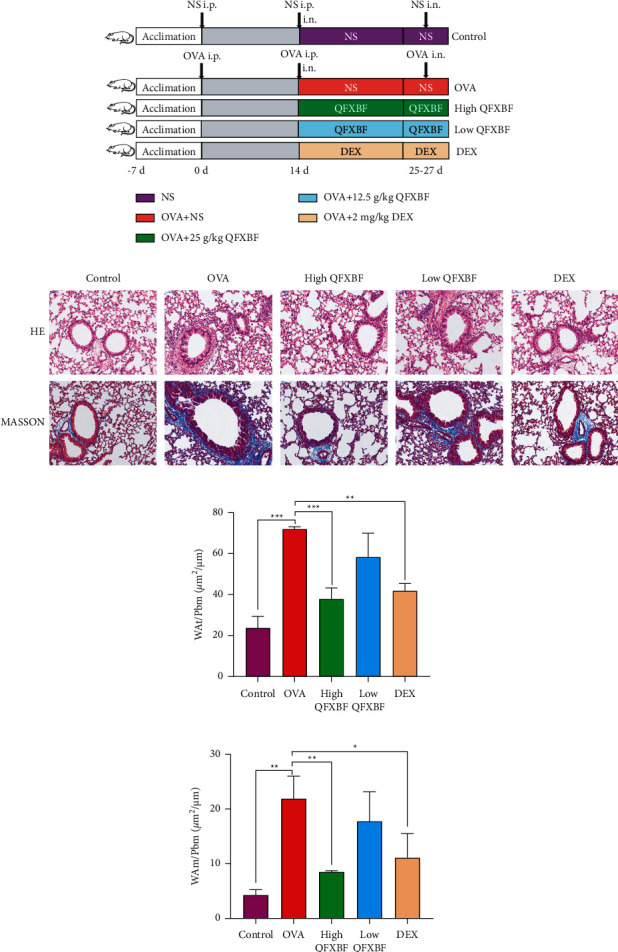
QFXBF inhibited the inflammatory condition and airway remodeling process of OVA-induced asthmatic mice. (a) Animal experimental protocol of this study. (b) Representative images of the mice lung tissue by HE and Masson staining. Scale bar, 50 *μ*m. (c) Quantitative analyses of WAt/Pbm. (d) Quantitative analyses of WAm/Pbm. All data are presented as means ± SD.  ^*∗*^*p* < 0.05,  ^*∗*^ ^*∗*^*p* < 0.01, and  ^*∗*^ ^*∗*^ ^*∗*^*p* < 0.001.

To verify the protective effects of QFXBF in the airway remodeling process of asthma, an OVA-induced asthmatic mouse model was established (Figure 5(a)). As shown in Figure 5(b), the airway mucosa epithelium and alveolar structure of the mice were relatively complete in the control group, and there were no significant inflammatory conditions in the lung tissues, whereas infiltration of peribronchial and perivascular inflammatory cells was observed in the OVA group; however, treatment with high QFXBF or DEX significantly alleviated the inflammatory conditions and infiltration of the immune cells, while the curative effects were not obvious in the low QFXBF group. Furthermore, QFXBF effectively alleviated the remodeling of the airway structure. Finally, collagen deposition was examined via Masson trichrome staining (Figure 5(b)), and overexpression of collagen was observed in the OVA group, while QFXBF significantly decreased the deposition of collagen. Compared with the control group, thickening of the bronchiolar wall and airway smooth muscle was observed in the lung tissue of the OVA mice, while treatment with high QFXBF and DEX alleviated the condition (Figures 5(c)–5(d)).

### 3.6. QFXBF Inhibits the Expression of *α*-SMA and PCNA in Lung Tissue

**Figure 6:**
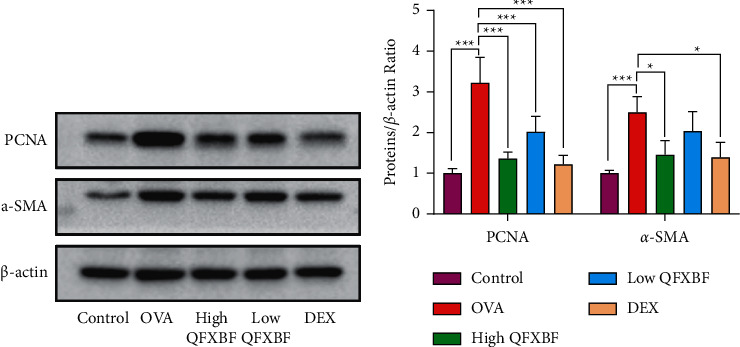
QFXBF inhibited the expression of *α*-SMA and PCNA in lung tissue. (a) Protein expressions of PCNA and *α*-SMA in lung tissue by western blot assay. (b) Quantification of results in (a). All data are presented as means ± SD.  ^*∗*^*p* < 0.05,  ^*∗*^ ^*∗*^*p* < 0.01, and  ^*∗*^ ^*∗*^ ^*∗*^*p* < 0.001.

In addition, we detected the expression of *α*-SMA and PCNA via western blotting. Compared with the control group, the protein expression of PCNA and *α*-SMA was significantly increased (*p* < 0.05) in the OVA group. Meanwhile, administration of high QFXBF and DEX significantly inhibited the protein expression of PCNA and *α*-SMA (Figures 6(a) and 6(b)).

### 3.7. Effects of QFXBF on Airway Remodeling Were Regulated by MEK/ERK Signaling Pathway

**Figure 7:**
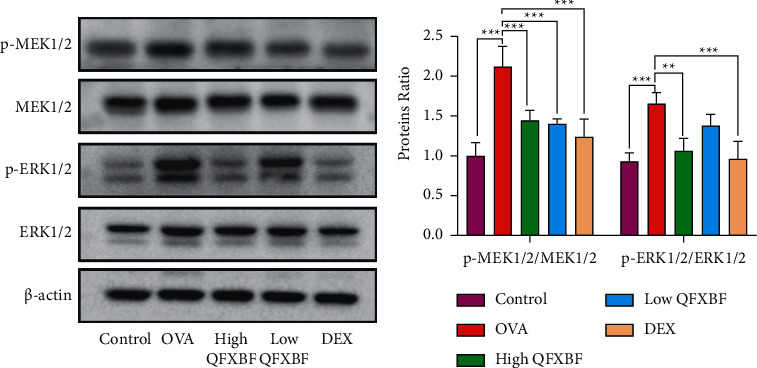
The effects of QFXBF on airway remodeling were regulated by MEK/ERK signaling pathway. (a) Protein expressions of p-MEK1/2, MEK1/2, p-ERK1/2, and ERK1/2 in lung tissue of the mice by western blot. (b) Quantification of results in (a). All data are presented as means ± SD. ^*∗*^*P* < 0.05, ^*∗∗*^*P* < 0.01, and ^*∗∗∗*^*P* < 0.001.

In order to determine whether QFXBF could affect MEK/ERK signaling during the process of OVA-induced airway remodeling, the protein expressions of p-MEK1/2, MEK1/2, p-ERK1/2, and ERK1/2 in mice treated with different concentrations of QFXBF were examined. As shown in Figures 7(a) and 7(b), the expressions of p-MEK1/2 and p-ERK1/2 in the lung tissues of the mice were markedly increased in the model group compared with the control group, while treatment with high QFXBF and DEX significantly decreased the protein expression of p-MEK1/2 and p-ERK1/2.

## 4. Discussion

Airway remodeling is an important pathological feature of asthma [18] and is also an important cause of repeated asthma attacks, causing the hyperresponsiveness of the airway and the chronic decline of lung function. Airway remodeling is described as the pathological reorganization of cells and molecular components of the airway wall, including airway epithelial detachment, goblet cell proliferation, increased mucus gland secretion, subepithelial fibrosis, hyperproliferation, and hypertrophy of ASMCs [19–21]. In these pathological changes, the proliferation of ASMCs has been recognized as one of the most important factors related to in vitro airway hyperresponsiveness and asthma severity [22]. Therefore, it is vital to develop potential drugs that could inhibit the proliferation of ASMCs. Traditional Chinese medicine (TCM) has a long history of the treatment of asthma with high therapeutic efficacy [23]. As a TCM for the treatment of asthma in China for many years, QFXBF has shown remarkable curative effects for the treatment and prevention of asthma. The results of previous studies indicated that QFXBF could alleviate the inflammatory condition in an asthma model [15]. However, little is known about the effects of QFXBF on airway remodeling. Our study demonstrated that treatment with QFXBF inhibited the proliferation of airway smooth muscle cells in vitro and airway remodeling in vivo. Our data also confirmed that the protective effects of QFXBF were affected by MEK/ERK signaling pathways in vitro and in vivo.

Previous studies have shown that the proliferation of ASMCs is the main characteristic of airway remodeling in asthma [24–26], which induces the remodeling process by increasing the thickness of the bronchial wall and airway smooth muscle [27, 28]. In this study, we explored the effects of QFXBF on the proliferation of ASMCs in vitro and in vivo. There are many inducers of airway smooth muscle proliferation, including growth factors, cytokines, and inflammatory mediators [29]. PDGF is known as the major stimulus that induces the proliferation of ASMCs [30] and participates in the process of airway remodeling [31–33]. Therefore, PDGF is often used to induce the excessive proliferation of ASMCs. Our results suggest that QFXBF inhibits the PDGF-induced hyperproliferation of ASMCs in a dose-dependent manner. Liu et al. reported that the thickness of the bronchial wall and airway smooth muscle was decreased in OVA-induced mice, while treatment with emodin alleviated this condition [16]. In this study, we demonstrated that the thickness of the bronchial walls and increased airway smooth muscles in OVA-induced mice were alleviated in the high QFXBF group in asthma animal models. Our results suggest that QFXBF is a potentially effective alternative therapy for improving airway remodeling.

PCNA is an indicator of cell proliferation status, and it is also an accessory protein of a polymerase that is necessary for DNA replication [34], which plays an important role in the initiation of cell proliferation [35]. *α*-SMA is a primary marker of airway smooth muscle. It is considered a crucial index for airway remodeling [36] and can significantly affect the contractility of airway smooth muscle [37]. The content of *α*-SMA is minimal in less-differentiated smooth muscle cells but opposite in differentiated and mature smooth muscle cells [38]. It has been reported that the herbal medicine FXF could inhibit the expression levels of *α*-SMA and PCNA in the OVA-challenged rat model, which indicates the potential therapeutic effect of FXF on airway remodeling in allergic airway disease [39]. In our study, the expressions of PCNA and *α*-SMA were used to examine the effects of QFXBF on airway smooth muscle proliferation. Our results also showed that the expressions of PCNA and *α*-SMA were increased in the lung tissues of mice asthma models and the hyperproliferative ASMCs, while QFXBF could alleviate the overexpression of *α*-SMA and PCNA. These results indicate that QFXBF exerts therapeutic effects on ASMC proliferation.

To further explore the underlying mechanisms of QFXBF in airway remodeling, we investigated the changes in the MEK/ERK signaling pathway following different treatments. The MEK/ERK signaling pathway plays a vital role in the pathogenesis and development of asthma and is known to aggravate inflammatory conditions and the airway remodeling process. As an important part of the MAPK signaling pathway, ERK-induced signaling can be transmitted from the extracellular region to the nucleus, which could further affect the proliferation of ASMCs and participate in the airway remodeling process in the development of asthma [40]. Shenmai injection has been reported to improve airway remodeling by regulating the ERK signaling pathway and affecting the proliferation of ASMCs [41]. Moreover, vasoactive intestinal peptides have been reported to inhibit the proliferation of airway smooth muscle cells in a mouse model of asthma via regulation of ERK1/2 signaling [42]. Our results are consistent with those of previous studies. In our study, the phosphorylation of MEK and ERK in OVA-induced mice and PDGF-induced ASMCs was markedly increased, indicating that the activation of the MEK/ERK signaling pathway is closely related to the development of asthma. Furthermore, U0126, a MEK/ERK signaling pathway inhibitor, not only reversed the PDGF-induced proliferation of ASMCs but also suppressed the activation of the MEK/ERK signaling pathway. Therefore, PDGF could have played an important role in the proliferation of ASMCs through the activation of MEK and ERK. We found that QFXBF dose-dependently attenuated the hyperproliferation of ASMCs and the expression of phosphorylated MEK/ERK. The underlying mechanism of the antiproliferative effects of QFXBF on PDGF-induced ASMCs and OVA-stimulated mice could be attributed to the inhibition of the MEK/ERK signaling pathway.

However, our study has certain limitations. Due to the complexity and diversity of the chemical composition of QFXBF, further studies should aim to identify which biologically active compounds of QFXBF protect against airway remodeling in asthmatic mice, which will be helpful to fully elucidate the antiasthmatic mechanisms of QFXBF.

## 5. Conclusions

In this study, we identified the protective role of QFXBF in asthma. QFXBF can inhibit the proliferation of ASMCs by suppressing the activation of MEK/ERK signaling in OVA-stimulated mice and PDGF-treated ASMCs, eventually ameliorating the process of airway remodeling. Our findings provide new evidence for the use of QFXBF as a potential antiremodeling candidate for the treatment of asthma.

## Data Availability

The datasets used and analyzed during the current study are available from the corresponding author on reasonable request.

## Abbreviations

QFXBF: Qufeng Xuanbi formula
DEX: Dexamethasone
OVA: Ovalbumin
H&E: Hematoxylin and eosin
ASMCs: Airway smooth muscle cells
ERK: Extracellular signal-regulated kinase
PDGF: Platelet-derived growth factor
Pbm: Bronchial basement membrane perimeter
WAt: Bronchial wall area
WAm: Bronchial smooth muscle area
TCM: Traditional Chinese medicine.

## Supplementary Materials

Supplementary Materials Supplementary File 1: representative ingredients of QFXBF. Supplementary File 2: original western blot images.
