# Supplementary material for: Qufeng Xuanbi Formula Ameliorates Airway Remodeling in Asthmatic Mice by Suppressing Airway Smooth Muscle Cell Proliferation through MEK/ERK Signaling Pathway
Source: Evid Based Complement Alternat Med. 2022 Feb 9;2022:1525110. doi: 10.1155/2022/1525110 (PMC8849894; doi:10.1155/2022/1525110)

Repeat 1

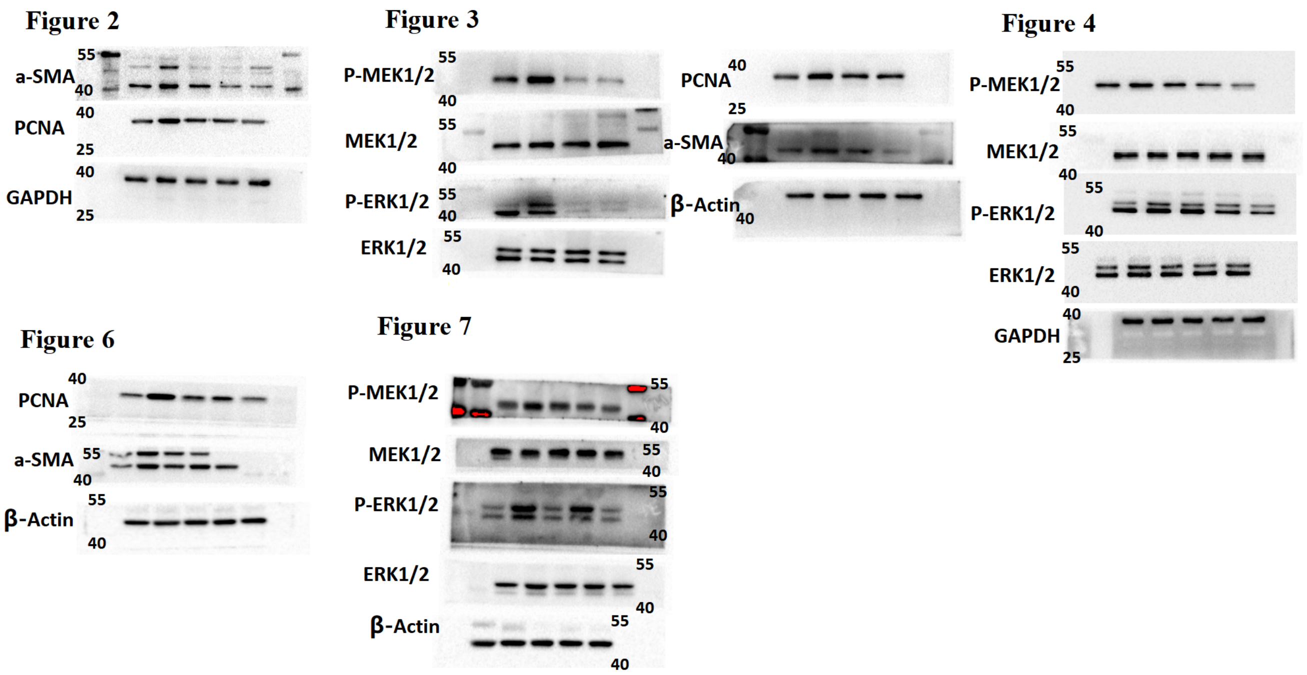

Repeat 2

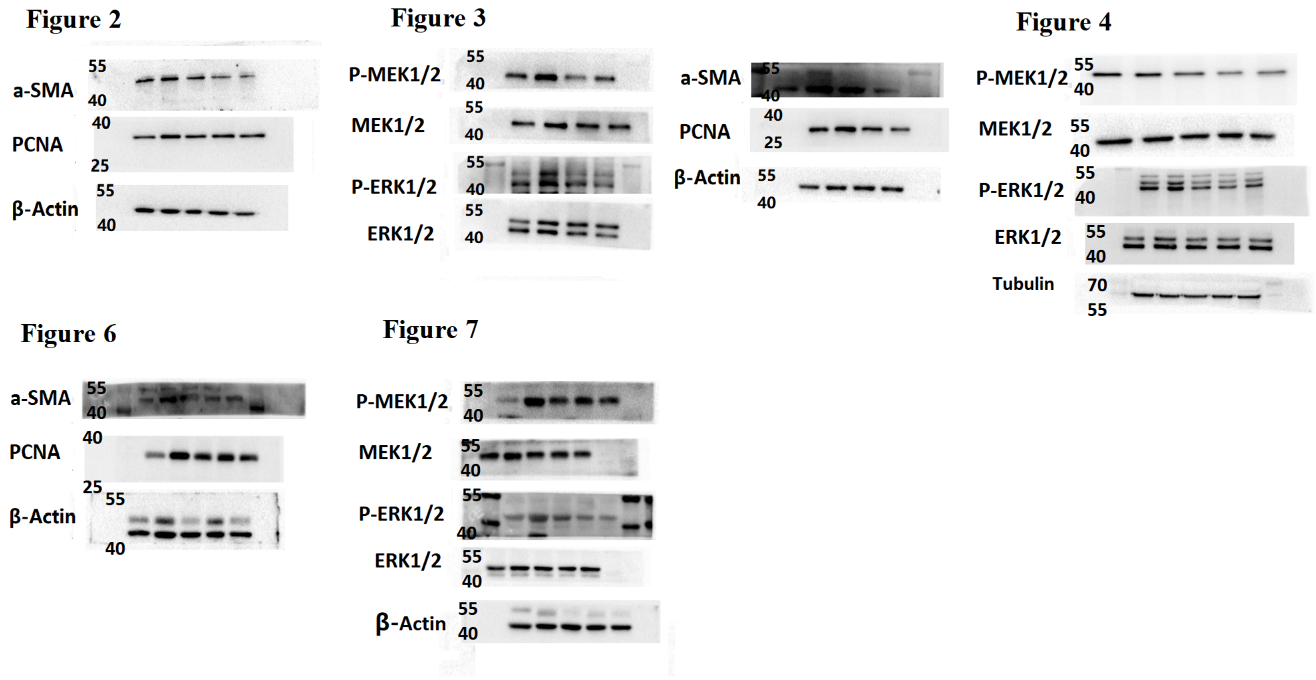

Repeat 3

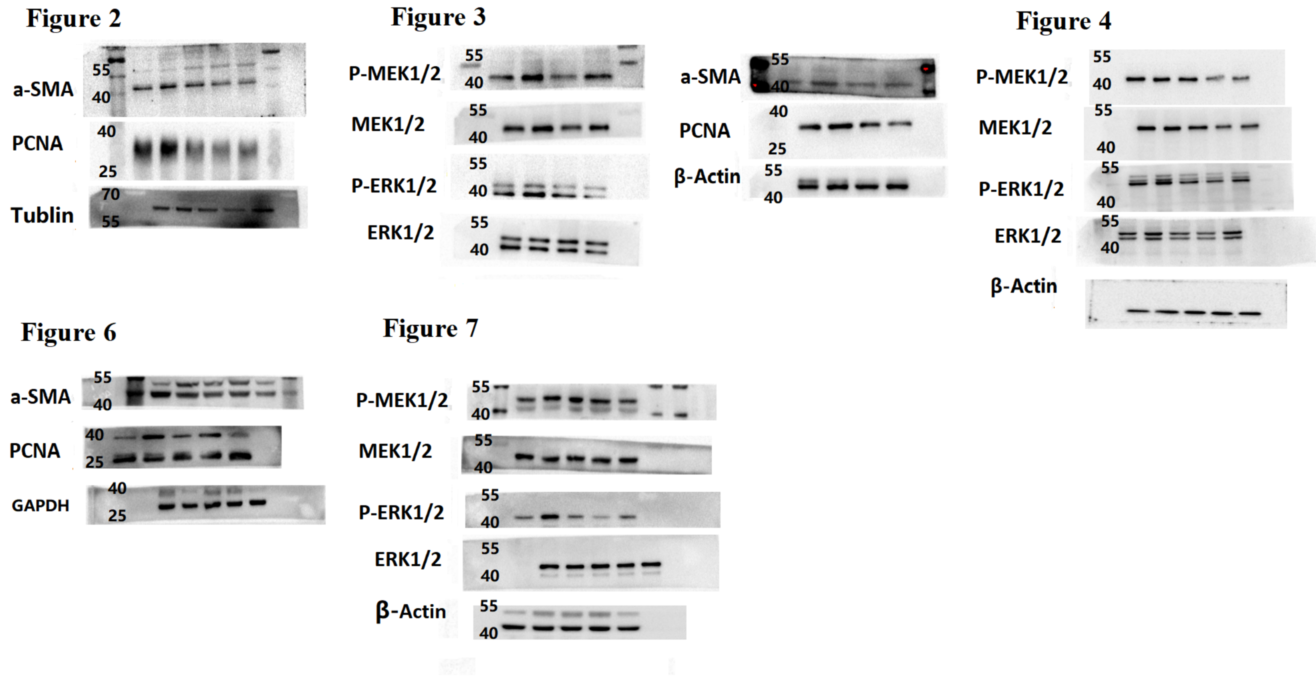

Supplement: Supplementary Materials — Supplementary File 1: representative ingredients of QFXBF. Supplementary File 2: original western blot images. [file 1525110.f1.zip › 1525110.f1/originalwesternblot images.pdf]
