# Supplementary material for: Qufeng Xuanbi Formula Ameliorates Airway Remodeling in Asthmatic Mice by Suppressing Airway Smooth Muscle Cell Proliferation through MEK/ERK Signaling Pathway
Source: Evid Based Complement Alternat Med. 2022 Feb 9;2022:1525110. doi: 10.1155/2022/1525110 (PMC8849894; doi:10.1155/2022/1525110)
Supplement: Supplementary Materials — Supplementary File 1: representative ingredients of QFXBF. Supplementary File 2: original western blot images. [file 1525110.f1.zip › 1525110.f1/Representative ingredients of QFXBF.pdf]

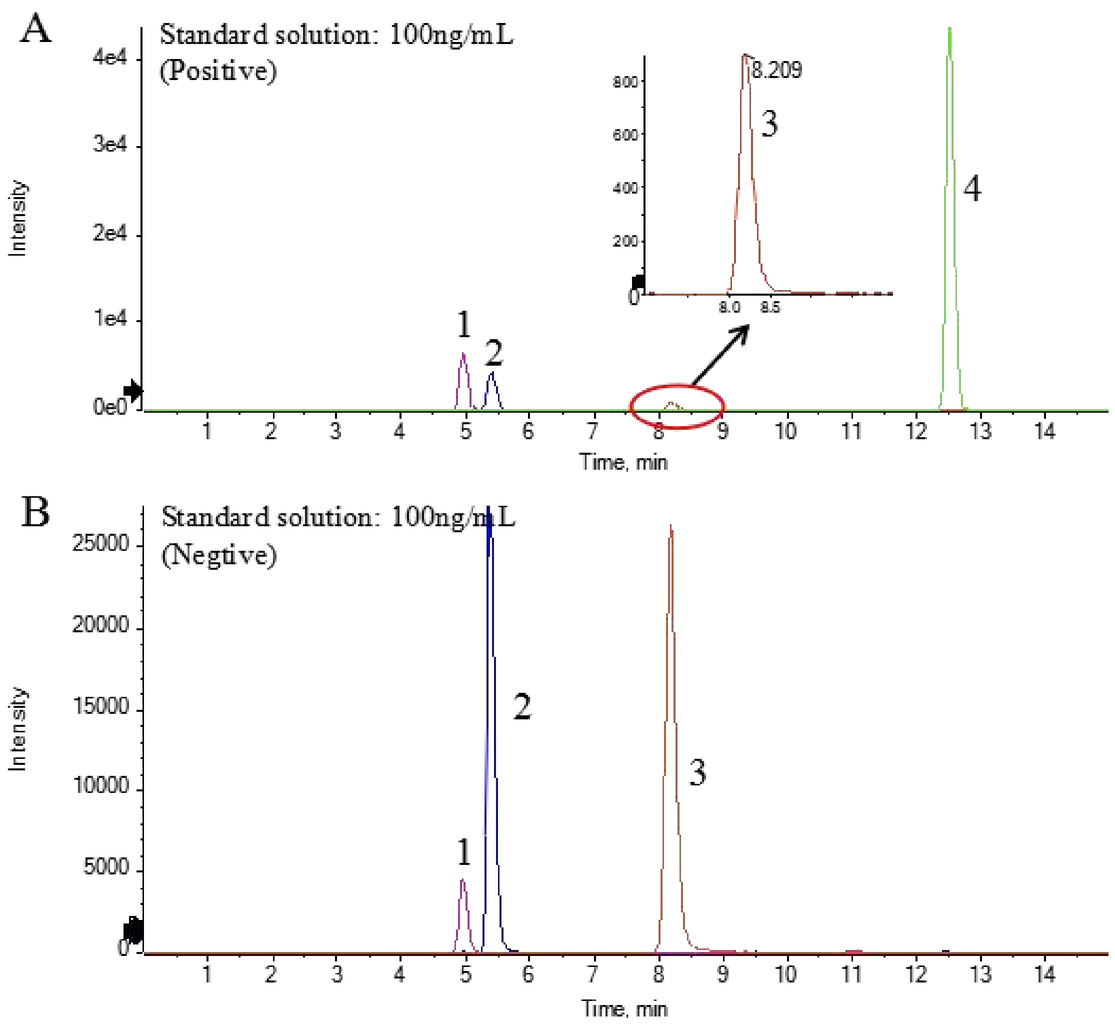

Figure 1. Extraction ion flow diagram of representative compound standard in QFXBF

A is positive ion mode, B is negative ion mode. 1, Amygdalin; 2, Iridoid; 3, Glycyrrhizic acid; 4, Osthol.

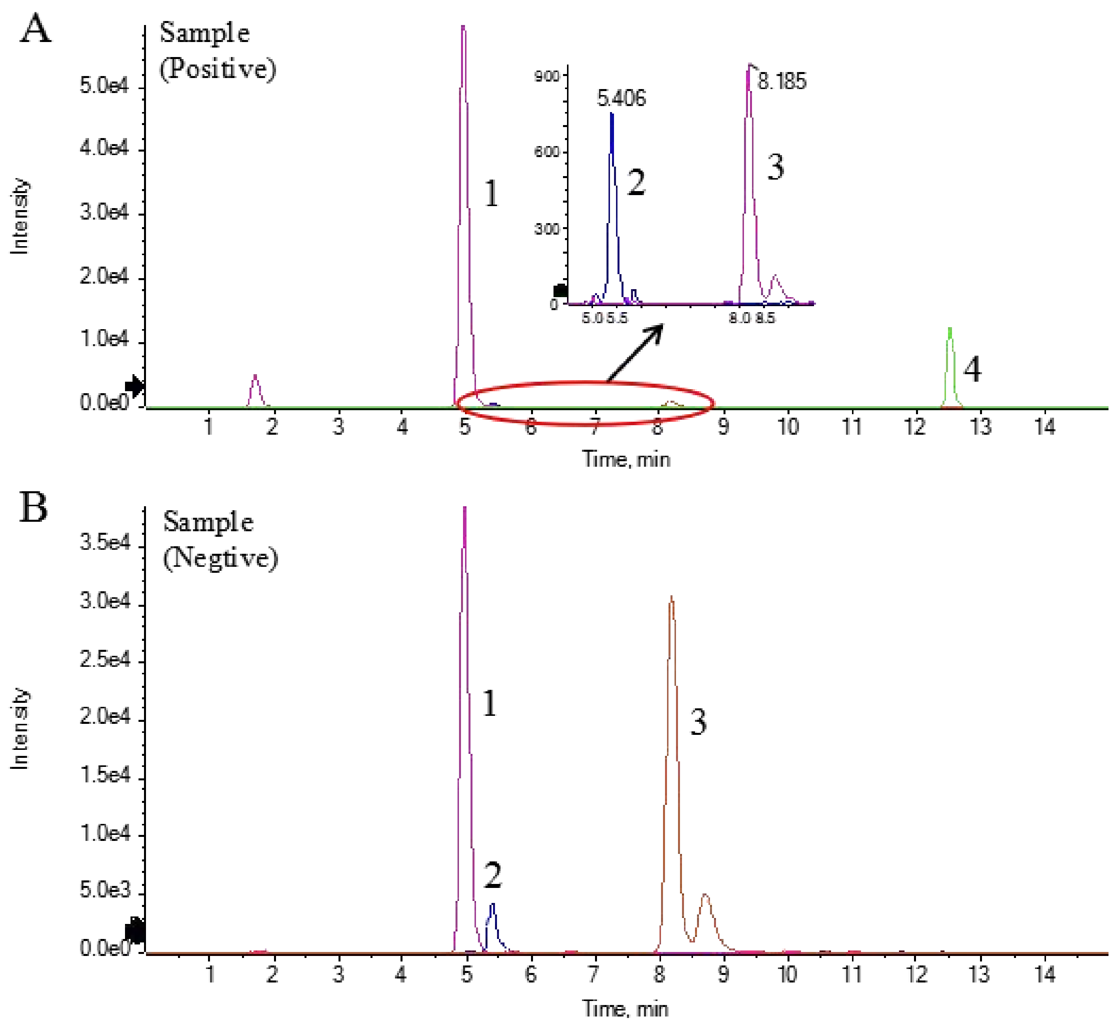

Figure 2. Extraction ion flow diagram of representative compounds in QFXBF (diluted 1000 times of mother liquor)

A is positive ion mode, B is negative ion mode. 1. Amygdalin; 2. Iridoid; 3. Glycyrrhizic acid; 4. Osthol.
